# Development and validation of an interpretable machine learning model for predicting incident gestational hypothyroidism using clinical laboratory markers

**DOI:** 10.3389/fmed.2026.1881157

**Published:** 2026-07-01

**Authors:** Liran Shen, Ru Liu, Yunbiao Zhang, Qingkai Wang, Zhiqiang Zhang, Mengting Li

**Affiliations:** 1 Department of Medical Laboratory Center, Shanxian Central Hospital, Heze, China; 2 Shantou Hospital of Traditional Chinese Medicine, Guangzhou University of Traditional Chinese Medicine, Shantou, Guangdong, China; 3 Chuzhou Hospital of Integrated Traditional Chinese and Western Medicine, Chuzhou, China; 4 Chuzhou First People’s Hospital, Chuzhou, China

**Keywords:** gestational hypothyroidism, LightGBM, machine learning, risk prediction, SHAP

## Abstract

**Objective:**

Traditional risk factors have limited performance for early identification of gestational hypothyroidism (GHT), and evidence remains limited on prediction using clinical laboratory markers.

**Methods:**

This single-center retrospective observational study included 407 pregnant women without GHT at baseline, including 164 with incident GHT and 243 without GHT during follow-up. Candidate predictors were extracted from electronic medical records and laboratory information systems. Least Absolute Shrinkage and Selection Operator (LASSO) regression, the Boruta algorithm, and Random Forest variable-importance ranking were used for feature selection. Twelve ML models were developed and compared. Model performance was evaluated using the area under the receiver operating characteristic curve (AUC), classification metrics, calibration curves, decision curve analysis (DCA), clinical impact curves, and residual analysis. Shapley Additive Explanations (SHAP) was used for interpretation.

**Results:**

Nine predictors were retained: zinc (Zn), iron (Fe), copper (Cu), calcium (Ca), vitamin D (vitD), vitamin E (vitE), albumin (ALB), alanine aminotransferase (ALT), and alkaline phosphatase (ALP). In the validation set, Lasso had the highest AUC [0.918; 95% confidence interval (CI), 0.871–0.964]. Light Gradient Boosting Machine (LightGBM) had a similar AUC (0.916; 95% CI, 0.866–0.965) and achieved the highest accuracy, positive predictive value (PPV), specificity, F1 score, and Youden’s J statistic. LightGBM also showed acceptable calibration performance in the internal validation set, clinical net benefit, and residual distribution; therefore, it was selected as the final model. SHAP identified Zn, ALP, ALB, Cu, vitE, ALT, Fe, Ca, and vitD as the leading contributors.

**Conclusion:**

A LightGBM model based on clinical laboratory markers showed balanced performance for predicting incident GHT. External prospective validation is required before clinical implementation.

## Introduction

Gestational hypothyroidism (GHT) is a common endocrine disorder during pregnancy and includes overt hypothyroidism and subclinical hypothyroidism (1). Recent studies have reported a disease burden of thyroid dysfunction during pregnancy across regions; however, prevalence estimates vary by region, iodine status, timing of testing, and diagnostic criteria (2). Thyroid dysfunction during pregnancy is not only a laboratory abnormality. It is also associated with maternal and fetal outcomes. A prospective cohort study reported an association between maternal thyroid dysfunction and increased risk of adverse pregnancy outcomes (3). Studies using large population databases also reported higher risks of pregnancy, delivery, and neonatal complications among pregnant women with hypothyroidism (4). Therefore, early identification and risk stratification of GHT remain clinical issues in prenatal care.

The diagnosis of GHT currently relies mainly on thyroid function markers, including thyroid-stimulating hormone (TSH) and free thyroxine (FT4). However, traditional high-risk factors have limited performance in early screening and risk stratification. An individual participant data meta-analysis of 25 cohorts and 65,559 pregnant women reported that selective screening based on currently recommended risk factors covered 58% of the population and detected only 59% of overt and subclinical hypothyroidism cases. A prediction model based on age, body mass index (BMI), smoking status, parity, and gestational week at the time of blood sampling yielded an area under the curve (AUC) of 0.58–0.63 (5). Another systematic review included 81 studies and assessed 36 candidate risk factors. The review found substantial heterogeneity in the evidence for most recommended risk factors and did not identify new clinical risk factors that consistently improved screening performance (6). Furthermore, the benefit of treating subclinical hypothyroidism during pregnancy remains uncertain. A meta-analysis of 11 randomized controlled trials involving 2,749 patients reported that levothyroxine treatment reduced the risk of pregnancy loss, with a risk ratio (RR) of 0.69 and a 95% confidence interval (CI) of 0.52–0.91, but did not significantly increase the live birth rate (RR, 1.01; 95% CI, 0.99–1.03) (7). Consequently, GHT management should shift from post-diagnostic intervention toward early risk prediction and individualized monitoring.

Nutritional, metabolic, and environmental factors regulate thyroid homeostasis during pregnancy. Iodine is a substrate for thyroid hormone synthesis. In a study of 559 pregnant women, serum iodine concentration was associated with FT4 levels across pregnancy stages, and morning urinary iodine concentration identified high TSH and low TSH with AUCs of 0.688 and 0.753, respectively (8). Studies in early pregnancy also reported insufficient urinary iodine in some pregnant women despite adequate iodine nutrition at the population level. These findings support the use of pregnancy-specific reference ranges for thyroid function assessment (9). Iron status is also associated with thyroid function during pregnancy. A systematic review and meta-analysis of 47 studies involving 53,152 pregnant women reported higher TSH levels and lower FT4 levels in women with iron deficiency than in those with sufficient iron. Hemoglobin was negatively correlated with TSH and positively correlated with FT4 (10). Nutritional evidence further indicates that iodine, selenium, iron, zinc, copper, magnesium, vitamin A, and other micronutrients participate in thyroid hormone synthesis and regulation (11). In addition, during pregnancy, copper, magnesium, zinc, iron, and other metal elements are associated with maternal thyroid function and metabolic status. Vitamin D status and heavy metal exposure may also affect thyroid homeostasis during pregnancy through immune regulation, oxidative stress, or endocrine disruption (12–14). Thyroid function is also linked to metabolic abnormalities during pregnancy. A prospective cohort study of 26,742 pregnant women reported that elevated TSH in early pregnancy was associated with increased risk of subsequent gestational diabetes mellitus (15). Taken together, these findings indicate that GHT-related risk may reflect a combined imbalance involving nutrition, trace elements, metabolism, and environmental exposure.

**FIGURE 1:**
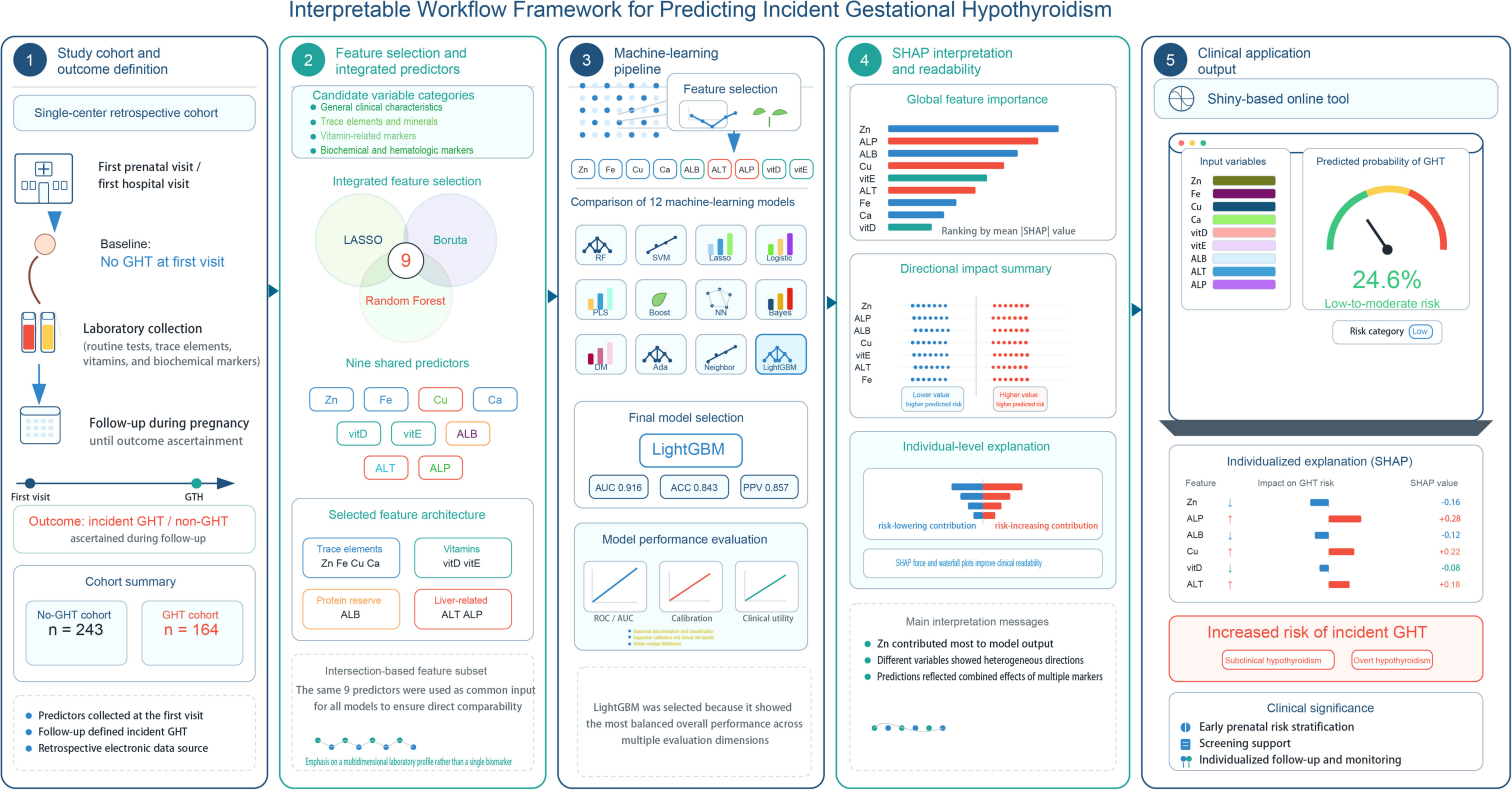
Graphical overview and program workflow of the study. The workflow summarizes data extraction, participant screening, missing-data handling, feature selection, model development and validation, SHAP-based interpretation, and Shiny-based deployment.

When multidimensional clinical variables and laboratory markers contribute to disease occurrence, traditional linear statistical models may not fully capture nonlinear associations or potential interactions among variables (16). Machine learning methods can integrate high-dimensional features, identify nonlinear associations, and model complex interactions. Shapley Additive Explanations (SHAP) can quantify the direction of contribution, relative importance, and potential threshold effects of each feature on the model output, thereby improving model readability and interpretability in clinical settings. For risk identification during pregnancy, this framework combines prediction and interpretation and may be more suitable for clinical decision support than traditional univariable or linear methods (17, 18). In recent years, machine learning models have been increasingly used for prediction, management, and pathophysiological assessment of pregnancy-related diseases, including gestational diabetes mellitus, preeclampsia, preterm birth, and fetal growth restriction (19–21). Previous studies have examined GHT in relation to prevalence, risk factors, nutritional status, and pregnancy outcomes. However, evidence remains limited on the prediction of incident GHT using clinical laboratory markers. Therefore, this study included pregnant women who first visited Shanxian Central Hospital between 2021 and 2025 and had no GHT diagnosis at baseline. The objective was to develop and validate a machine-learning predictive model based on multidimensional laboratory indicators and to apply SHAP for model interpretation, thereby supporting early risk assessment and individualized monitoring of GHT. The graphical overview and program workflow of the study are presented in Figure 1, summarizing the transition from data extraction and participant screening to missing-data handling, feature selection, model development, model interpretation, and Shiny-based deployment.

## Materials and methods

### Study design, participants, and outcome ascertainment

This single-center retrospective study was designed for prediction model development and internal validation. De-identified electronic medical records and laboratory information system data were used to retrospectively screen pregnant women who had their first eligible prenatal visit between 2021 and 2025. The first eligible visit was defined as the earliest visit during the current pregnancy at which the participant did not meet the diagnostic criteria for GHT and had the clinical and laboratory variables required for model development.

A total of 407 pregnant women with available post-baseline thyroid function records or prenatal follow-up information sufficient for outcome ascertainment were included in the final analytic dataset, including 164 who developed incident GHT and 243 who did not. The observed event fraction of 40.3% reflects the outcome distribution of the analytic dataset and was not intended to estimate the population incidence of incident GHT among unselected pregnant women. All data were obtained from the hospital’s electronic medical record and laboratory information systems. After extraction, all study data were de-identified and used only for statistical analysis and model development. Given the retrospective design and absence of additional intervention, informed consent was waived. The study was approved by the Medical Ethics Committee of Shanxian Central Hospital (approval number: LL 2026022).

The GHT group included overt hypothyroidism and subclinical hypothyroidism. According to current clinical guidelines for thyroid disease during pregnancy, overt hypothyroidism was defined as elevated TSH with decreased FT4, and subclinical hypothyroidism was defined as elevated TSH with FT4 within the reference range. In this study, GHT was defined as TSH > 4.20 μIU/mL after the first eligible visit, with FT4 < 12 pmol/L for overt hypothyroidism and FT4 within 12–22 pmol/L for subclinical hypothyroidism (22). Outcome status was determined from available retrospective post-baseline records. Incident GHT was defined as the first post-baseline thyroid function result that met the diagnostic criteria for overt or subclinical hypothyroidism. Participants were classified as non-GHT if no available post-baseline thyroid function record or prenatal follow-up information met the diagnostic criteria for GHT.

The inclusion criteria were as follows: (1) completion of thyroid function testing and concurrent clinical laboratory tests required for model development at the first visit during the current pregnancy, without meeting the diagnostic criteria for GHT at that visit; (2) availability of subsequent thyroid function retesting or clinical follow-up data sufficient to determine whether incident GHT occurred after the first visit; (3) availability of baseline clinical data and laboratory measurements required for model development; and (4) for participants with multiple visit records, retention of only the first record that met the inclusion criteria and contained complete data. The exclusion criteria were as follows: (1) confirmed thyroid disease before pregnancy or long-term use of thyroid-related treatment, including levothyroxine; (2) fulfillment of the diagnostic criteria for GHT at the first visit; (3) comorbid conditions that could affect thyroid function, trace element metabolism, nutritional status, or inflammatory status, including severe hepatic or renal dysfunction, hematologic diseases, autoimmune diseases, and malignant tumors; (4) acute infection, systemic inflammatory response, or other acute stress states; and (5) substantial missingness in key variables or inability to determine the follow-up outcome.

### Candidate predictors and missing-data handling

All study variables were extracted from the electronic medical record system and laboratory information system at the first visit. Candidate predictors were selected according to clinical relevance, availability in the hospital electronic medical record and laboratory information systems, and prior evidence. These variables included general clinical characteristics, trace elements and minerals, vitamin-related markers, biochemical metabolic markers, and hematologic markers. General clinical characteristics included age and gestational week (GW) at the first visit. Trace elements and minerals included zinc (Zn), iron (Fe), copper (Cu), calcium (Ca), phosphorus (P), and magnesium (Mg). Vitamin-related markers included vitamin A (vitA), vitamin D (vitD), vitamin E (vitE), and vitamin D3 (vitD3). Biochemical metabolic markers included albumin (ALB), alanine aminotransferase (ALT), alkaline phosphatase (ALP), total bile acid (TBA), urea (UREA), glucose (GLU), cholyglycine (CG), aspartate aminotransferase (AST), creatinine (CREA), uric acid (UA), lactate dehydrogenase (LDH), and gamma-glutamyl transferase (GGT). Hematologic markers included white blood cell count (WBC), neutrophil count (NEUT), lymphocyte count (LYMPH), red blood cell count (RBC), platelet count (PLT), mean corpuscular volume (MCV), mean corpuscular hemoglobin (MCH), and mean corpuscular hemoglobin concentration (MCHC).

Missing data patterns were assessed before imputation and are summarized in Supplementary Table S1. A total of 72 participants had at least one missing value, corresponding to 111 missing values in the original dataset. The highest missing proportion was observed for phosphorus (P; 4.18%), followed by lactate dehydrogenase (LDH; 3.44%) and zinc (Zn; 3.44%). Among the final retained predictors, missingness ranged from 0 to 3.44%. Missing values were handled using multiple imputation by chained equations (MICE), with five imputed datasets generated using a fixed random seed of 123. Multiple imputation was used to reduce sample loss, loss of statistical power, and potential selection bias from complete-case analysis. The imputed data were then used for subsequent feature selection, model development, and validation analyses. To reduce information leakage, the training and validation sets were first separated, and preprocessing procedures, including imputation and standardization, were implemented within the modeling workflow.

### Feature selection strategy

To reduce redundancy and potential multicollinearity among candidate predictors and improve model stability, this study used a feature selection strategy that combined Least Absolute Shrinkage and Selection Operator (LASSO) regression, the Boruta algorithm, and random forest variable-importance ranking. This combined strategy was chosen to reduce reliance on a single feature-selection algorithm and to identify predictors with stable contributions across both penalized regression and tree-based importance measures. All feature selection procedures were performed in the training set.

First, LASSO regression was applied to candidate variables in the training set using the glmnet package. A penalty term was imposed on the regression coefficients to perform variable shrinkage and dimensionality reduction. The optimal penalty parameter λ was determined using 10-fold cross-validation. Variables with coefficients shrunk to zero were excluded. Subsequently, the Boruta algorithm, based on the random forest framework, was applied to the candidate features to assess variable importance. This algorithm constructs shadow features and compares the importance distributions of real and shadow features to identify variables with stable discriminative value across repeated training. In parallel, a random forest model was used to compute importance scores for candidate variables and rank them by their contribution to the classification task. Finally, the results from LASSO regression, the Boruta algorithm, and random forest variable-importance ranking were integrated to define the core feature subset for subsequent machine-learning model development. A Spearman correlation heatmap was generated to assess the correlation structure among the selected features and to examine whether marked clusters of high correlation were present.

### Model development and evaluation

After feature selection, the final feature subset was used as input variables to develop machine learning models for predicting incident GHT. The final analytic dataset was divided into a training set and an internal validation set at an approximate 7:3 ratio. The training set included 286 participants, comprising 115 women with incident GHT and 171 women without GHT. The internal validation set included 121 participants, comprising 49 women with incident GHT and 72 women without GHT.

The evaluated models included RandomForest, support vector machine with kernel function (SVM_Kernel), logistic regression model (LogisticModel), neighbor-based method (NeighborMethod), partial least squares model (PLSModel), BoostingMethod, neural network model (NeuralNet), Bayesian model (BayesMethod), DiscriminantModel, Least Absolute Shrinkage and Selection Operator model (Lasso), Adaptive Boosting, and Light Gradient Boosting Machine (LightGBM). To ensure comparability across models, all models were trained and evaluated using the same data partition, input features, and preprocessing workflow. For threshold-dependent performance metrics, including accuracy, PPV, sensitivity, specificity, F1 score, Youden’s J statistic, and NPV, predicted probabilities were converted into binary classifications using a prespecified probability cutoff of 0.5. Participants with predicted probabilities ≥ 0.5 were classified as GHT, whereas those with predicted probabilities < 0.5 were classified as non-GHT. No class weighting, oversampling, undersampling, SMOTE, or other resampling strategy was applied during model training. Model discrimination was assessed using the receiver operating characteristic (ROC) curve and the AUC, with 95% CIs calculated for the AUC. Classification performance was evaluated using accuracy, positive predictive value (PPV), sensitivity, specificity, F1 score, Youden’s J statistic, and negative predictive value (NPV). Model calibration was assessed using calibration curves and quantitative calibration metrics, including the Brier score, calibration intercept, and calibration slope. Potential clinical utility was assessed using decision curve analysis (DCA) and clinical impact curves. Prediction error distributions were further examined through residual analysis, including reverse-cumulative absolute residual curves and residual box plots. The final model was selected according to overall performance rather than validation AUC alone, with consideration of discrimination, threshold-dependent classification performance, calibration, clinical net benefit, residual distribution, and interpretability.

### SHAP-based model interpretation and visualization

To improve the interpretability of the final prediction model, SHAP was used to interpret model outputs. SHAP was selected because it provides both global and individual-level explanations, allowing the relative importance, direction, and magnitude of each predictor’s contribution to be visualized. SHAP analysis quantified the direction and magnitude of each input feature’s contribution to the predicted outcome and assessed the relative role of each variable in the prediction process. First, the mean absolute SHAP value for each variable was computed to assess global feature importance, and the variables were ranked accordingly. A SHAP summary plot was generated to display the distribution of feature values and the direction of their effects on model output. SHAP dependence plots were then generated to examine the relationship between individual variables and model output. At the individual level, SHAP waterfall and force plots were used to present the composition of the prediction for a single sample and to visualize the positive or negative contribution of each variable to the predicted value.

### Statistical analysis

Basic statistical analyses and model-related calculations were performed using SPSS version 27.0 and R version 4.5.1. The main R packages used for model development and feature selection included lightgbm version 4.6.0, Boruta version 8.0.0, and glmnet version 4.1–10. SHAP-based interpretation analyses were performed in Python version 3.12 using the shap package version 0.51.0. Continuous variables with a normal distribution were reported as mean ± standard deviation (SD) and compared between groups using the independent-samples *t*-test. Continuous variables with non-normal distributions were reported as medians and interquartile ranges (IQRs) and compared between groups using the Wilcoxon rank-sum test. Categorical variables were reported as n (%) and compared between groups using the χ^2^ test or Fisher’s exact test, as appropriate. All statistical tests were two-sided, and *P* < 0.05 was considered statistically significant. For baseline characteristics in Table 1, *P*-values were reported as descriptive unadjusted comparisons, and no multiple-comparison correction was applied. These baseline comparisons were not used as the sole basis for predictor selection.

**TABLE 1:** Baseline characteristics.

Variables	Total (*N* = 407)	No-GHT (*N* = 243)	GHT (*N* = 164)	*P*-value
Age (years)	30.00 (27.00, 34.00)	30.00 (27.00, 34.00)	30.00 (27.00, 34.00)	0.603
GW (weeks)	10.00 [9.00, 12.00]	10.00 [8.00, 12.00]	11.00 (9.00, 13.00)	< 0.001
Zn (μmol/L)	114.52 ± 19.69	119.81 ± 18.33	106.68 ± 19.06	< 0.001
Fe (mmol/L)	8.93 ± 1.02	9.15 ± 0.96	8.60 ± 1.03	< 0.001
Cu (μmol/L)	24.46 ± 4.71	23.35 ± 4.50	26.11 ± 4.53	< 0.001
Ca (mmol/L)	2.27 ± 0.08	2.29 ± 0.07	2.25 ± 0.07	< 0.001
P(mmol/L)	1.02 ± 0.10	1.04 ± 0.09	1.01 ± 0.10	0.004
Mg (mmol/L)	0.84 (0.81, 0.87)	0.85 (0.82, 0.87)	0.84 (0.80, 0.86)	0.003
vitA (ng/mL)	0.36 ± 0.06	0.37 ± 0.06	0.35 ± 0.05	< 0.001
vitD (ng/mL)	40.57 ± 6.73	41.75 ± 6.58	38.83 ± 6.57	< 0.001
vitE (ng/mL)	6.37 ± 1.05	6.54 ± 1.03	6.10 ± 1.03	< 0.001
vitD3 (pg/mL)	34.15 ± 5.21	34.72 ± 5.10	33.32 ± 5.29	0.008
ALB (g/L)	40.65 ± 2.94	41.32 ± 2.77	39.67 ± 2.92	< 0.001
ALT (U/L)	14.90 (11.00, 18.45)	14.00 (10.30, 17.05)	16.40 (13.17, 19.83)	< 0.001
ALP (U/L)	69.50 (56.95, 80.90)	64.70 (52.45, 75.60)	76.55 (66.18, 89.43)	< 0.001
TBA (μmol/L)	2.34 (1.86, 2.91)	2.34 (1.85, 2.92)	2.33 (1.90, 2.89)	0.950
UREA (mmol/L)	2.88 ± 0.60	2.86 ± 0.63	2.91 ± 0.56	0.429
GLU (mmol/L)	4.73 ± 0.56	4.73 ± 0.58	4.73 ± 0.52	0.988
CG (μg/mL)	3.48 ± 0.91	3.47 ± 0.87	3.50 ± 0.96	0.689
AST (U/L)	18.29 ± 4.08	17.86 ± 4.02	18.92 ± 4.10	0.010
CREA (μmol/L)	45.28 ± 6.87	45.38 ± 6.73	45.13 ± 7.09	0.716
UA (μmol/L)	214.89 ± 30.12	211.59 ± 29.37	219.78 ± 30.63	0.007
LDH (U/L)	148.60 (135.95, 162.95)	145.40 (132.40, 159.60)	154.05 (143.05, 169.48)	< 0.001
GGT (U/L)	11.97 ± 3.09	11.66 ± 3.11	12.43 ± 3.01	0.014
WBC ( × 10^9^/L)	8.66 ± 1.22	8.45 ± 1.18	8.98 ± 1.21	< 0.001
NEUT ( × 10^9^/L)	5.60 ± 1.00	5.37 ± 0.93	5.94 ± 1.00	< 0.001
LYMPH ( × 10^9^/L)	1.91 ± 0.38	1.95 ± 0.38	1.85 ± 0.39	0.010
RBC ( × 10^12^/L)	4.28 ± 0.34	4.29 ± 0.33	4.26 ± 0.35	0.512
PLT ( × 10^9^/L)	270.16 ± 33.20	274.27 ± 33.33	264.07 ± 32.15	0.002
MCV (fL)	87.71 ± 4.86	87.64 ± 5.02	87.82 ± 4.64	0.724
MCH (pg)	29.94 ± 1.81	29.97 ± 1.79	29.90 ± 1.85	0.707
MCHC (g/L)	338.00 (331.00, 345.00)	337.00 (330.00, 343.00)	341.00 (333.00, 347.00)	0.003

GW, gestational week; Zn, zinc; Fe, iron; Cu, copper; Ca, calcium; P, phosphorus; Mg, magnesium; vitA, vitamin A; vitD, vitamin D; vitE, vitamin E; vitD3, vitamin D3; ALB, albumin; ALT, alanine aminotransferase; ALP, alkaline phosphatase; TBA, total bile acid; UREA, urea; GLU, glucose; CG, cholyglycine; AST, aspartate aminotransferase; CREA, creatinine; UA, uric acid; LDH, lactate dehydrogenase; GGT, gamma-glutamyl transferase; WBC, white blood cell count; NEUT, neutrophil count; LYMPH, lymphocyte count; RBC, red blood cell count; PLT, platelet count; MCV, mean corpuscular volume; MCH, mean corpuscular hemoglobin; MCHC, mean corpuscular hemoglobin concentration; GHT, gestational hypothyroidism.

## Results

### Patient characteristics

A total of 407 pregnant women were included in this study, comprising 243 in the No-GHT group and 164 in the GHT group. Age did not differ significantly between the two groups (*P* = 0.603). Compared with the No-GHT group, the GHT group had lower levels of Zn, Fe, Ca, P, Mg, vitA, vitD, vitE, vitD3, and ALB, and a higher level of Cu, with unadjusted *P* < 0.05. In addition, the GHT group had higher levels of ALT, ALP, AST, UA, LDH, GGT, WBC, NEUT, and MCHC, and lower levels of LYMPH and PLT than the No-GHT group, also with unadjusted *P* < 0.05. No significant differences were observed between the two groups in TBA, UREA, GLU, CG, CREA, RBC, MCV, or MCH (Table 1).

### Selection of modeling variables

**FIGURE 2:**
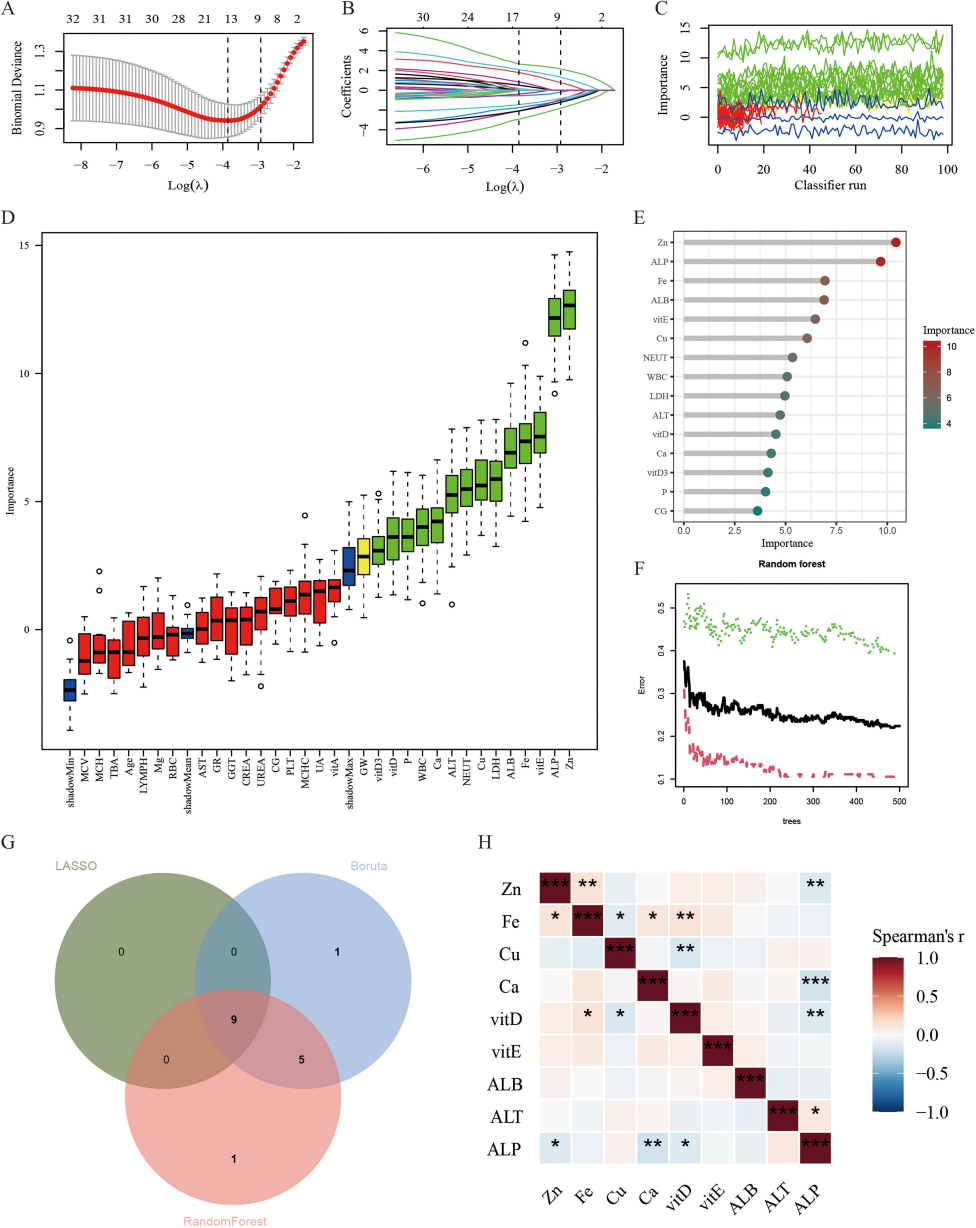
Variable selection and correlation analysis. **(A)** Ten-fold cross-validation curve of LASSO regression for determining the optimal penalty parameter λ. **(B)** LASSO coefficient path plot showing coefficient shrinkage across different λ values. **(C)** Variable importance trajectories across classifier runs in the Boruta algorithm. **(D)** Distribution and ranking of variable importance identified by the Boruta algorithm. **(E)** Variable importance ranking based on the random forest model. **(F)** Changes in random forest model error with increasing numbers of trees. **(G)** Venn diagram of variables selected by LASSO, Boruta, and random forest. **(H)** Spearman correlation heatmap of the final selected variables. *P < 0.05, **P < 0.01, and ***P < 0.001.

To improve the stability of variable selection and reduce selection bias associated with a single algorithm, this study combined LASSO regression, the Boruta algorithm, and random forest variable importance ranking for feature selection. The optimal penalty parameter λ in LASSO regression was determined by cross-validation. As λ increased, the regression coefficients gradually shrank, and 9 candidate variables were retained (Figures 2A,B). The Boruta algorithm identified variables with stable discriminative contribution by comparing the importance of original variables with that of shadow variables. Zn, ALP, Fe, and ALB ranked high in importance scores (Figures 2C,D). Random forest analysis ranked candidate variables by importance. To highlight the main discriminative variables, only the top 15 variables were presented. Zn and ALP ranked highest in importance (Figure 2E). The random forest error curve plateaued as the number of decision trees increased (Figure 2F). The intersection of the three feature selection methods yielded 9 core variables, including Zn, Fe, Cu, Ca, vitD, vitE, ALB, ALT, and ALP, which were then used in subsequent model development (Figure 2G). The correlation heatmap showed no pronounced clustering of high correlations among the selected variables (Figure 2H).

### Model development and performance assessment

Based on the final 9 selected variables, this study developed and compared 12 machine learning models: RandomForest, SVM_Kernel, LogisticModel, NeighborMethod, PLSModel, BoostingMethod, NeuralNet, BayesMethod, DiscriminantModel, Lasso, AdaptiveBoosting, and LightGBM.

**FIGURE 3:**
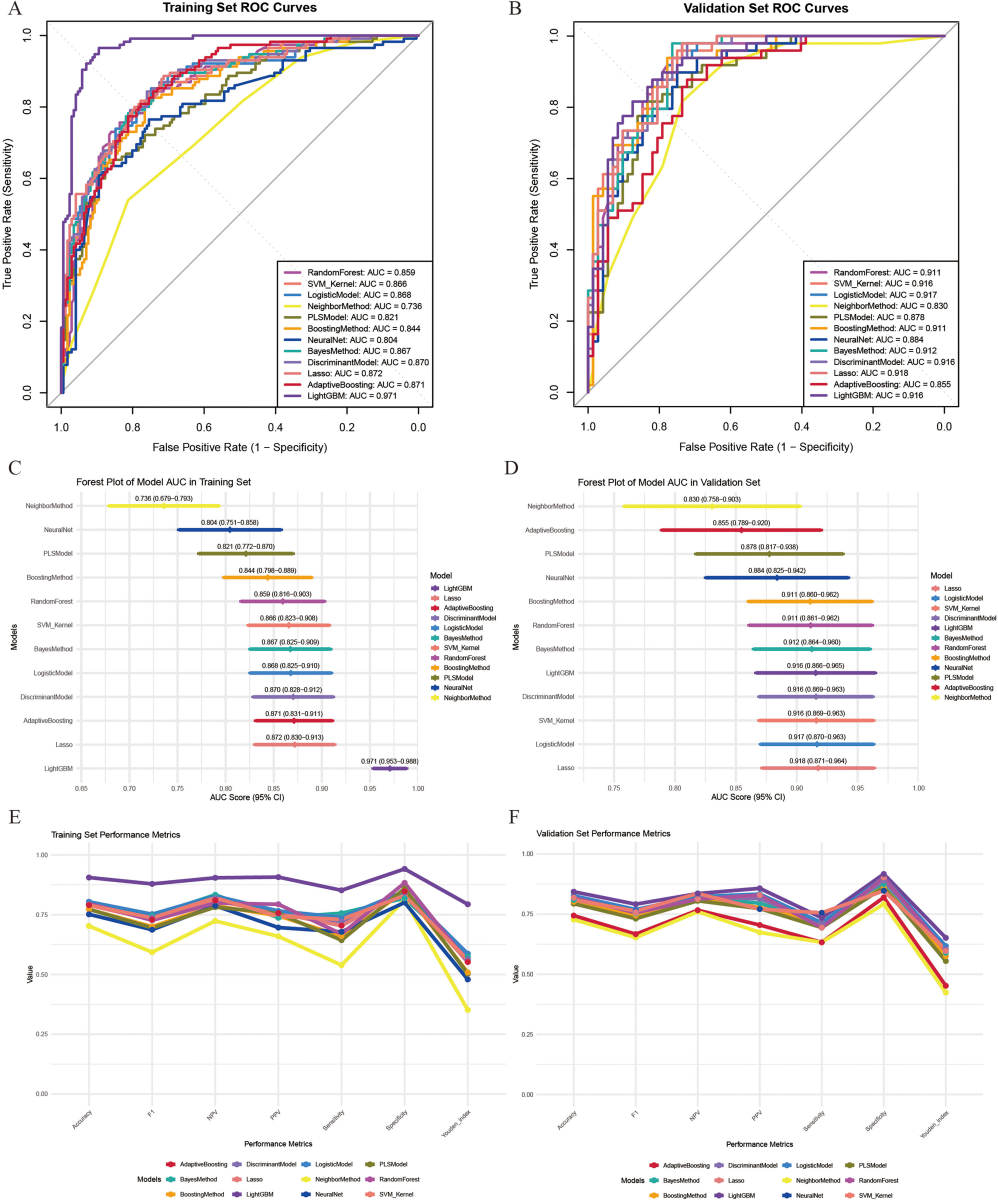
Discrimination and classification performance of machine learning models in the training and validation sets. **(A)** ROC curves in the training set. **(B)** ROC curves in the validation set. **(C)** Forest plot of AUC values in the training set. **(D)** Forest plot of AUC values in the validation set. **(E)** Comparison of classification performance metrics in the training set. **(F)** Comparison of classification performance metrics in the validation set.

In the training set, LightGBM yielded the highest overall performance, with an AUC of 0.971 (95% CI, 0.953–0.988). LightGBM also achieved the highest accuracy (0.906), PPV (0.907), sensitivity (0.852), specificity (0.942), F1 score (0.879), Youden’s J statistic (0.794), and NPV (0.904) (Figures 3A,C,E and Table 2). In the validation set, all models retained discriminatory ability. Lasso had the highest AUC of 0.918 (95% CI, 0.871–0.964), whereas LightGBM had a comparable AUC of 0.916 (95% CI, 0.866–0.965), with an absolute difference of 0.002 (Figures 3B,D). Although LightGBM did not yield the numerically highest AUC, it achieved the highest accuracy (0.843), PPV (0.857), specificity (0.917), F1 score (0.791), and Youden’s J statistic (0.651) in the validation set. Considering the negligible AUC difference and the more balanced threshold-dependent classification performance, LightGBM was retained for further comprehensive evaluation and interpretation (Figure 3F and Table 3).

**TABLE 2:** Performance of machine learning models in the training set.

Model	AUC (95% CI)	Accuracy	PPV	Sensitivity	Specificity	F1 score	Youden’s J	NPV
RandomForest	0.859 (0.816–0.903)	0.797	0.794	0.670	0.883	0.726	0.553	0.799
SVM_Kernel	0.866 (0.823–0.908)	0.790	0.743	0.730	0.830	0.737	0.561	0.821
LogisticModel	0.868 (0.825–0.910)	0.804	0.766	0.739	0.848	0.752	0.587	0.829
NeighborMethod	0.736 (0.679–0.793)	0.703	0.660	0.539	0.813	0.593	0.352	0.724
PLSModel	0.821 (0.772–0.870)	0.773	0.755	0.643	0.860	0.695	0.503	0.782
BoostingMethod	0.844 (0.798–0.889)	0.773	0.745	0.661	0.848	0.700	0.509	0.788
NeuralNet	0.804 (0.751–0.858)	0.752	0.696	0.678	0.801	0.687	0.479	0.787
BayesMethod	0.867 (0.825–0.909)	0.794	0.737	0.757	0.819	0.747	0.575	0.833
DiscriminantModel	0.870 (0.828–0.912)	0.794	0.755	0.722	0.842	0.738	0.564	0.818
Lasso	0.872 (0.830–0.913)	0.790	0.757	0.704	0.848	0.730	0.552	0.810
AdaptiveBoosting	0.871 (0.831–0.911)	0.790	0.757	0.704	0.848	0.730	0.552	0.810
LightGBM	0.971 (0.953–0.988)	0.906	0.907	0.852	0.942	0.879	0.794	0.904

AUC, area under the receiver operating characteristic curve; CI, confidence interval; PPV, positive predictive value; NPV, negative predictive value; SVM, support vector machine; PLS, partial least squares; Lasso, least absolute shrinkage and selection operator; LightGBM, light gradient boosting machine.

**TABLE 3:** Performance of machine learning models in the validation set.

Model	AUC (95% CI)	Accuracy	PPV	Sensitivity	Specificity	F1 score	Youden’s J	NPV
RandomForest	0.911 (0.861–0.962)	0.818	0.829	0.694	0.903	0.756	0.597	0.813
SVM_Kernel	0.916 (0.869–0.963)	0.810	0.771	0.755	0.847	0.763	0.602	0.836
LogisticModel	0.917 (0.870–0.963)	0.826	0.833	0.714	0.903	0.769	0.617	0.823
NeighborMethod	0.830 (0.758–0.903)	0.727	0.674	0.633	0.792	0.653	0.424	0.760
PLSModel	0.878 (0.817–0.938)	0.793	0.773	0.694	0.861	0.731	0.555	0.805
BoostingMethod	0.911 (0.860–0.962)	0.802	0.778	0.714	0.861	0.745	0.575	0.816
NeuralNet	0.884 (0.825–0.942)	0.810	0.771	0.755	0.847	0.763	0.602	0.836
BayesMethod	0.912 (0.864–0.960)	0.810	0.795	0.714	0.875	0.753	0.589	0.818
DiscriminantModel	0.916 (0.869–0.963)	0.818	0.814	0.714	0.889	0.761	0.603	0.821
Lasso	0.918 (0.871–0.964)	0.818	0.829	0.694	0.903	0.756	0.597	0.813
AdaptiveBoosting	0.855 (0.789–0.920)	0.744	0.705	0.633	0.819	0.667	0.452	0.766
LightGBM	0.916 (0.866–0.965)	0.843	0.857	0.735	0.917	0.791	0.651	0.835

AUC, area under the receiver operating characteristic curve; CI, confidence interval; PPV, positive predictive value; NPV, negative predictive value; SVM, support vector machine; PLS, partial least squares; Lasso, least absolute shrinkage and selection operator; LightGBM, light gradient boosting machine.

**FIGURE 4:**
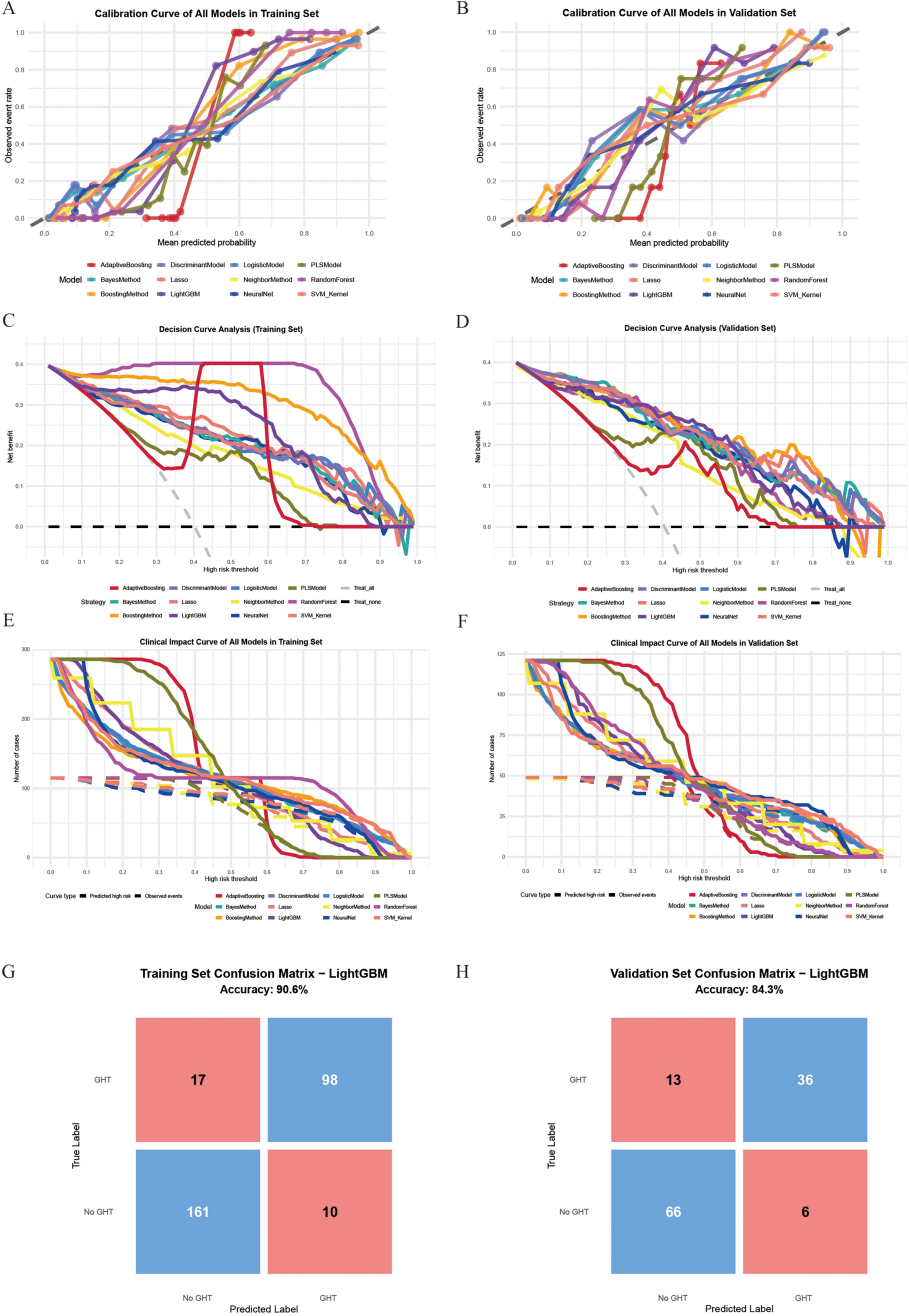
Calibration, clinical utility, and confusion matrices of the LightGBM model. **(A)** Calibration curves in the training set. **(B)** Calibration curves in the validation set. **(C)** Decision curve analysis in the training set. **(D)** Decision curve analysis in the validation set. **(E)** Clinical impact curves in the training set. **(F)** Clinical impact curves in the validation set. **(G)** Confusion matrix of the LightGBM model in the training set. **(H)** Confusion matrix of the LightGBM model in the validation set.

Calibration curves showed overall agreement between predicted probabilities and observed outcomes in the training and internal validation sets (Figures 4A,B). Quantitative calibration metrics are shown in Supplementary Table S2. In the internal validation set, DiscriminantModel, LogisticModel, and SVM_Kernel had relatively low Brier scores of 0.119, 0.119, and 0.120, respectively. For the final LightGBM model, the Brier score, calibration intercept, and calibration slope were 0.077, 0.022, and 2.198 in the training set, and 0.131, 0.096, and 1.040 in the internal validation set, respectively. These results indicated acceptable calibration performance of LightGBM in the internal validation set. DCA yielded positive net benefit for most models within specific threshold probability ranges (Figures 4C,D). Clinical impact curves indicated that the predicted number of high-risk cases decreased as the risk threshold increased, with a trend generally consistent with the number of observed events (Figures 4E,F). The LightGBM confusion matrices further supported its classification performance on both the training and validation sets (Figures 4G,H).

**FIGURE 5:**
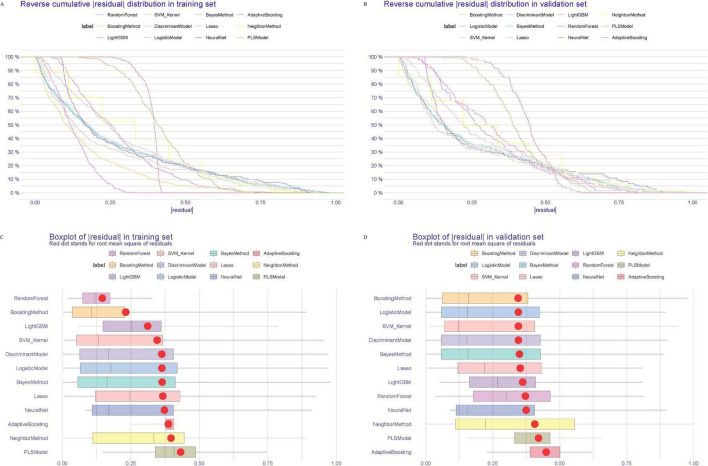
Residual distribution analysis of machine learning models. **(A)** Reverse cumulative absolute residual distribution in the training set. **(B)** Reverse cumulative absolute residual distribution in the validation set **(C)**. Boxplot of absolute residuals in the training set. **(D)** Boxplot of absolute residuals in the validation set.

Residual analysis was performed to assess the distribution of prediction errors. Reverse cumulative absolute residual curves and residual box plots demonstrated differences in residual distributions across models. LightGBM exhibited relatively low and stable residual levels in both the training and validation sets, with no marked clustering of large residuals (Figures 5A–D). Based on its comparable discrimination, balanced classification performance, acceptable calibration in the internal validation set, clinical net benefit, residual distribution, and compatibility with SHAP-based interpretation, LightGBM was selected as the final model for subsequent interpretation analysis.

In the GW-adjusted sensitivity analysis, the LightGBM model achieved an AUC of 0.977 in the training set and 0.915 in the internal validation set, which was comparable to the primary model. In the gain-based feature-importance ranking, ALP remained the leading predictor, whereas GW showed the lowest contribution among the included variables (Supplementary Table S3). These findings suggest that the predictive contribution of ALP was not fully explained by gestational week.

### SHAP-based model interpretation

**FIGURE 6:**
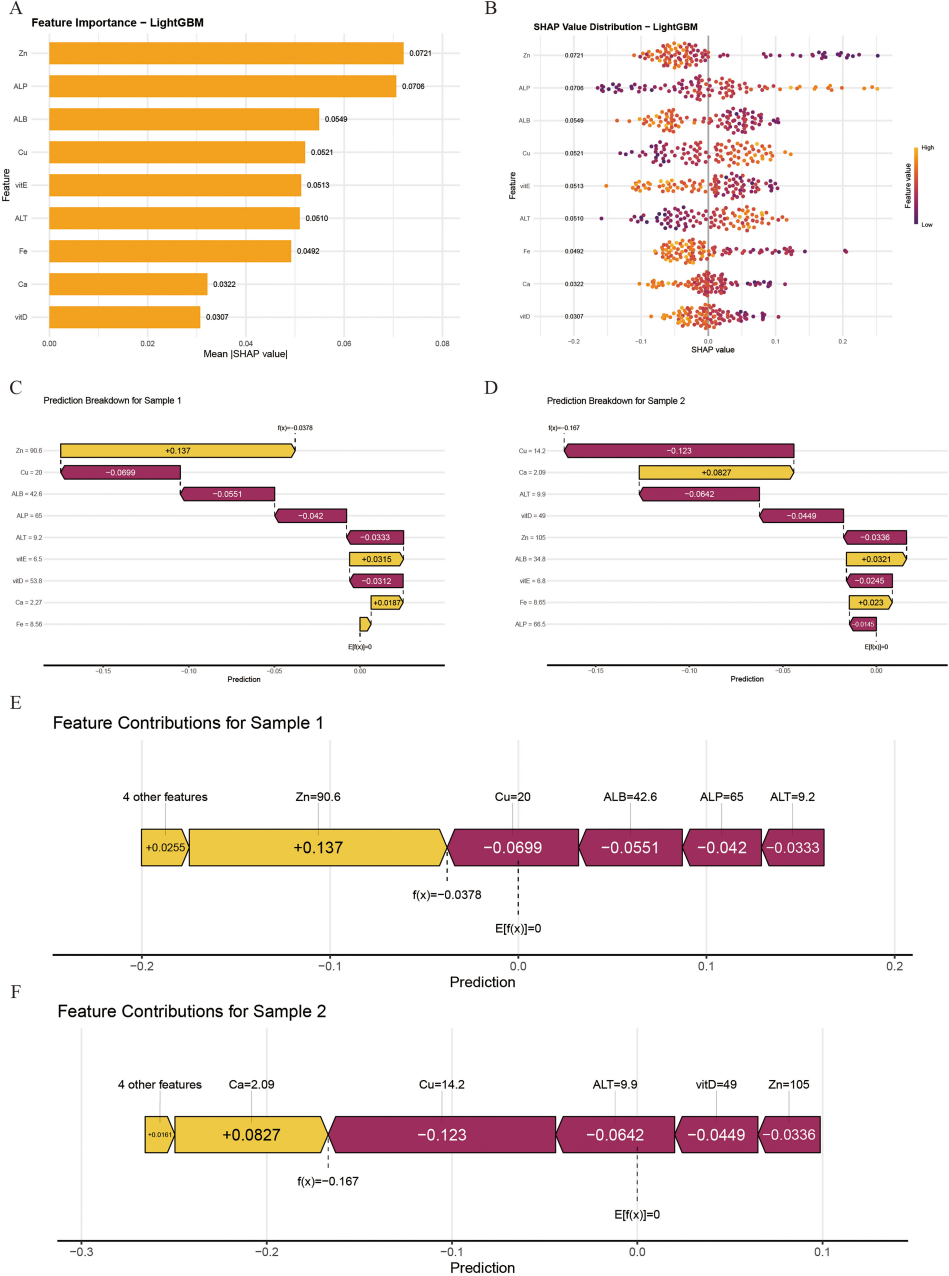
SHAP-based interpretation of the LightGBM model. **(A)** Variable importance ranking based on mean absolute SHAP values; **(B)** SHAP summary plot showing the distribution of feature values and their direction of contribution to model output; **(C)** SHAP waterfall plot for sample 1; **(D)** SHAP waterfall plot for sample 2; **(E)** SHAP force plot for sample 1, corresponding to the same individual shown in **(C)**; **(F)** SHAP force plot for sample 2, corresponding to the same individual shown in **(D)**.

To further interpret the predictions generated by LightGBM, this study used SHAP to quantify each variable’s contribution to the model’s output. Ranking by mean absolute SHAP value identified Zn as the largest contributor to model prediction, followed by ALP, ALB, Cu, vitE, ALT, Fe, Ca, and vitD (Figure 6A). The SHAP summary plot demonstrated heterogeneous directions of feature effects. Lower levels of Zn, ALB, Fe, Ca, vitD, and vitE generally shifted model output toward higher GHT risk. In contrast, higher levels of ALP, Cu, and ALT were generally associated with higher predicted risk (Figure 6B).

0

Individual-level SHAP interpretation demonstrated that model predictions were jointly determined by multiple variables rather than dominated by a single indicator. For sample 1, Zn made a positive contribution to the prediction, whereas Cu, ALB, ALP, and ALT mainly made negative contributions, resulting in a final model output of *f*(x) = -0.0378 (Figure 6C). The corresponding SHAP force plot displayed the cumulative direction and magnitude of each variable contribution for this sample (Figure 6E). For sample 2, Cu, ALT, vitD, and Zn mainly shifted the prediction in the negative direction. In contrast, Ca made a relatively strong positive contribution to model output, resulting in a final model output of *f*(x) = -0.167 (Figure 6D). The SHAP force plot for this sample was consistent with the waterfall plot and indicated that the prediction was also determined by the combined effects of multiple variables (Figure 6F).

**FIGURE 7:**
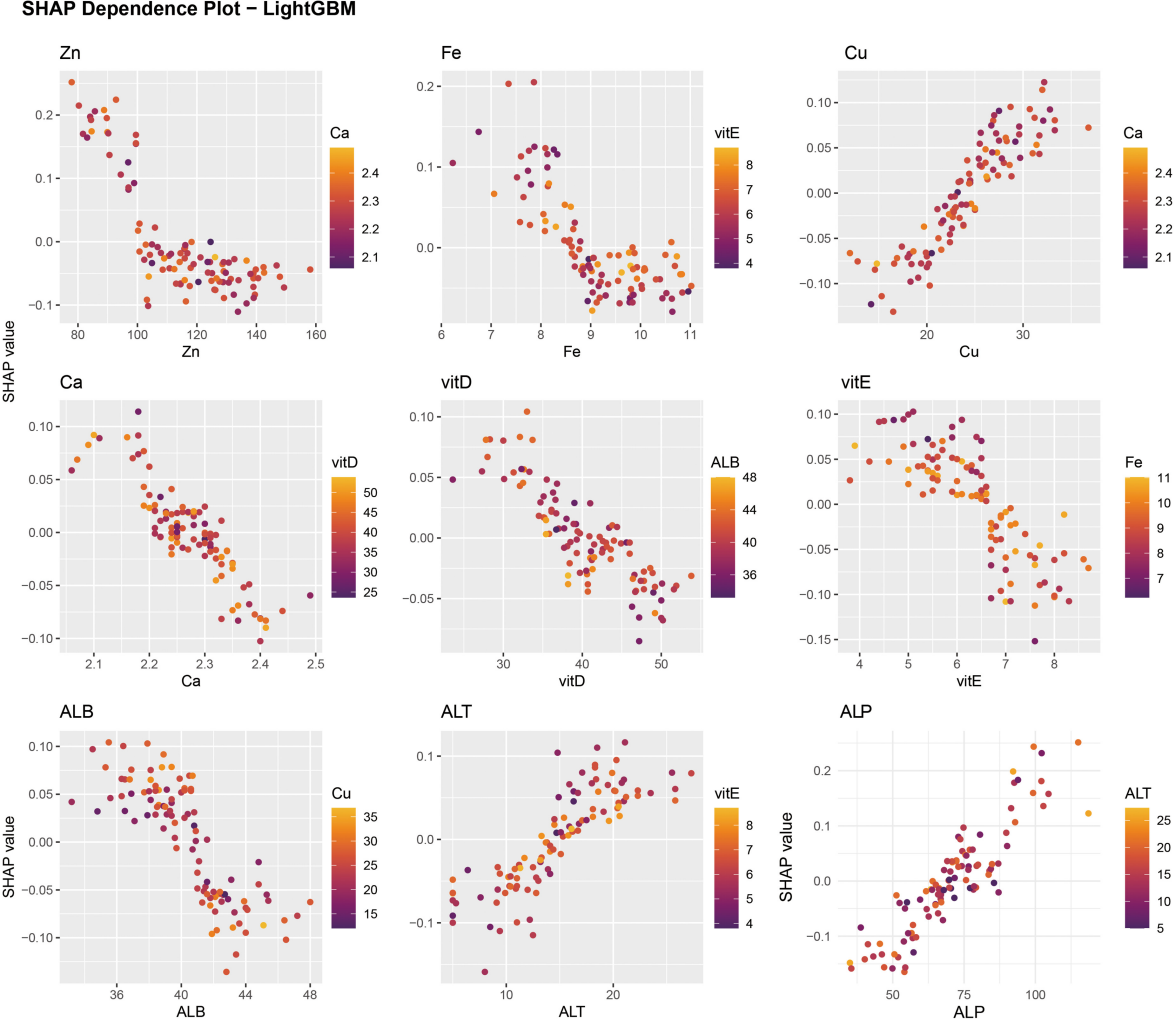
SHAP dependence plots of variables in the LightGBM model. The plots show the relationships between Zn, Fe, Cu, Ca, vitD, vitE, ALB, ALT, and ALP and their corresponding SHAP values. The x-axis represents the feature value, the y-axis represents the SHAP value, and the color scale indicates the interacting feature’s value, illustrating potential nonlinear associations between predictors and the model output.

SHAP dependence plots further identified nonlinear relationships between individual variables and model output. Zn, Fe, Ca, vitD, vitE, and ALB showed overall negative trends, with higher levels generally associated with lower SHAP values. In contrast, Cu, ALT, and ALP exhibited overall positive trends, with higher levels generally corresponding to higher SHAP values (Figure 7). These findings indicated that multivariable effects, nonlinearity, and directional heterogeneity characterized the GHT-related risk pattern captured by LightGBM.

### Model deployment and visualization applications

**FIGURE 8:**
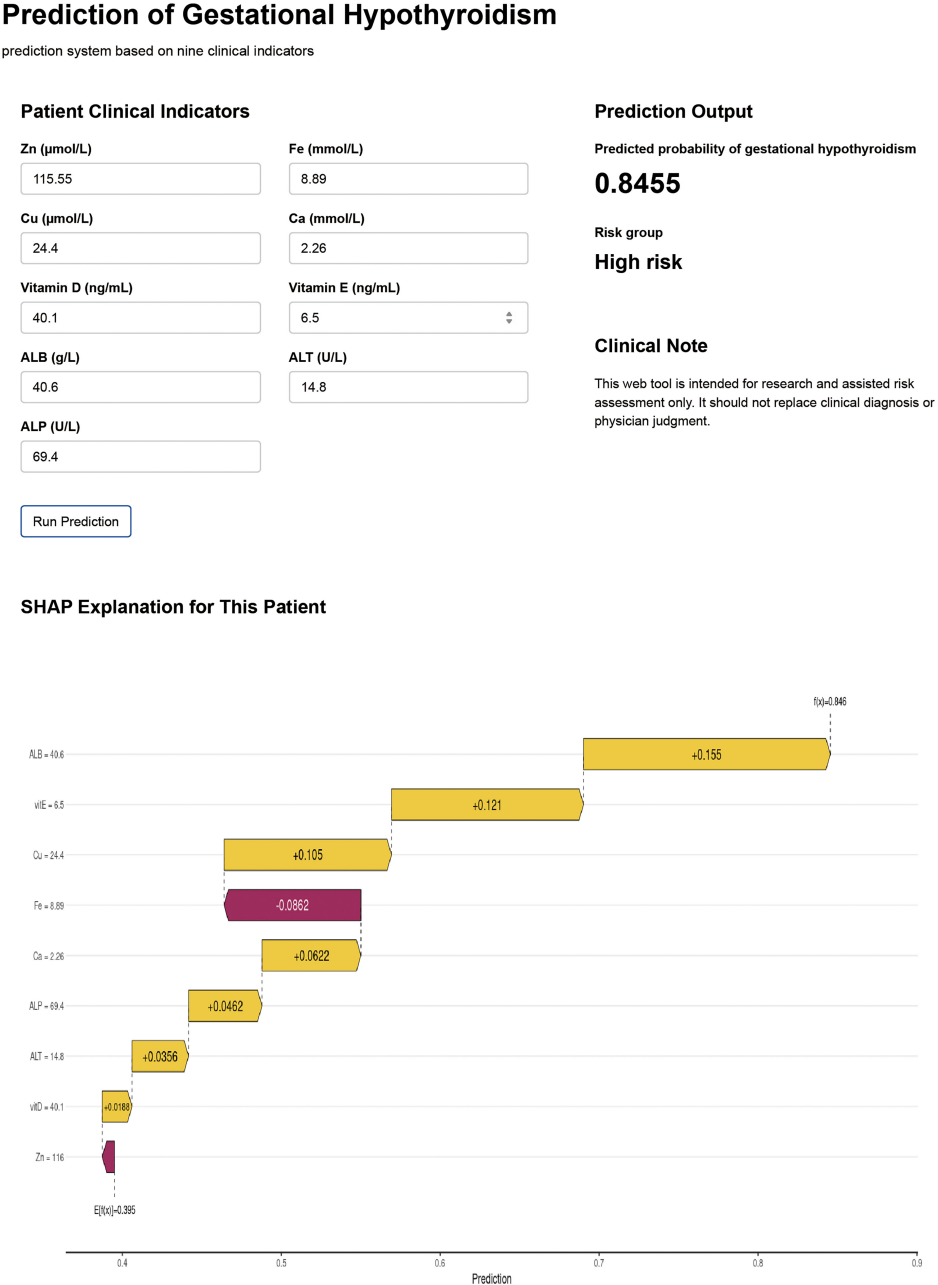
Web-based risk prediction calculator for GHT based on the LightGBM model. The Shiny-based calculator incorporates the nine variables in the final model: Zn, Fe, Cu, Ca, vitD, vitE, ALB, ALT, and ALP. After entering the corresponding clinical indicators, the system provides the individualized predicted probability of GHT and the corresponding risk classification in real time. A SHAP-based explanation plot is also provided to visualize the direction and magnitude of each variable’s contribution to the individual prediction.

To improve the accessibility and usability of LightGBM, this study deployed the model as a Shiny-based online risk prediction system. The system incorporated the 9 final model features, including Zn, Fe, Cu, Ca, vitD, vitE, ALB, ALT, and ALP. After entering the corresponding values, users can obtain the predicted probability of individual GHT risk and the corresponding risk stratification result in real time (Figure 8). The system also provides an individualized SHAP-based interpretation interface. This interface displays the direction and magnitude of each feature’s contribution to the current prediction, thereby presenting model output and interpretability results within the same workflow. The online tool has been released on ShinyApps.io at https://predicttheoutcome123.shinyapps.io/shiny_lgb_project/. Overall, this deployment provides a direct interface for individualized risk assessment and interpretability visualization based on LightGBM.

## Discussion

This study developed and validated machine learning models for predicting the risk of incident GHT. By integrating LASSO regression, the Boruta algorithm, and random forest variable importance ranking, 9 core variables were identified: Zn, Fe, Cu, Ca, vitD, vitE, ALB, ALT, and ALP. Among 12 models, LightGBM showed the best overall performance in the training set. In the internal validation set, Lasso had a slightly higher AUC than LightGBM; however, the absolute difference was only 0.002. LightGBM showed more balanced performance in accuracy, PPV, specificity, F1 score, and Youden’s J statistic, and it also demonstrated acceptable calibration in the internal validation set, clinical net benefit, and stable residual distribution. Therefore, LightGBM was selected as the final model based on overall performance rather than AUC alone. SHAP analysis further identified Zn as the largest contributor to model output, followed by ALP, ALB, Cu, vitE, ALT, Fe, Ca, and vitD. These findings suggest that incident GHT risk was captured by a multidimensional laboratory profile rather than by a single thyroid function marker. The profile involved trace element homeostasis, vitamin status, protein nutritional reserve, and liver-related metabolic markers.

Mineral-related variables occupied a central position in the final model. Gu et al evaluated the individual and combined effects of iodine, selenium, zinc, calcium, magnesium, and iron on thyroid hormones in 489 Chinese pregnant women and reported that Ca was positively associated with FT3, whereas Zn was positively associated with FT4. These findings indicate an association between mineral status and the thyroid hormone profile during pregnancy (23). A machine learning study using National Health and Nutrition Examination Survey (NHANES) data from 7,779 participants further identified nonlinear relationships between multiple mineral intakes and thyroid dysfunction. Ca, Zn, Mg, and related indicators were associated with a lower risk of thyroid dysfunction (24). The case-control study by Vargas-Uricoechea et al also reported that zinc deficiency was associated with higher frequencies of functional thyroid disease and thyroid autoimmunity (25). These findings are consistent with the direction observed in this study, in which lower levels of Zn, Fe, and Ca were associated with a higher predicted risk of GHT. At the mechanistic level, Zn and Fe are important regulators of thyroid peroxidase (TPO) activity. TPO is a key enzyme in thyroid hormone synthesis, catalyzing iodine oxidation and tyrosine iodination. Deficiency of Zn and Fe may affect TPO catalytic activity and reduce the efficiency of thyroid hormone synthesis (12, 26). Abnormal serum Ca may also contribute to the occurrence and development of GHT by affecting intracellular Ca^2+^ signaling in thyroid cells, particularly through iodine organification and thyroid hormone release mediated by the TSH downstream Gq/G11–PLC–Ca^2+^ pathway (27).

The inclusion of vitamin markers and metal elements further indicates that GHT risk is associated with nutritional status and oxidative stress. Zheng et al reported that vitamin D deficiency in early pregnancy was associated with increased risk of GHT, with an adjusted odds ratio (OR) of 1.32 (95% CI, 1.01–1.74) (28). Liu et al used NHANES data and reported that higher vitamin E intake was associated with lower prevalence of subclinical hypothyroidism and autoimmune thyroiditis. However, this association was mainly observed in men (29). These findings are generally consistent with the direction identified in this study, in which lower levels of vitD and vitE were associated with a higher predicted risk of GHT. However, they also indicate that the role of vitamin markers may vary across populations, measurement methods, and gestational stages. In addition, higher Cu levels in this study shifted model output toward higher GHT risk. Zhou et al reported that prenatal exposure to metal mixtures was associated with altered neonatal thyroid function in 2,444 mother-infant pairs (30). At the mechanistic level, Cu is a key cofactor for several antioxidant enzymes, including ceruloplasmin (CP) and superoxide dismutase (SOD). These enzymes participate in the clearance of reactive oxygen species (ROS) in thyroid cells. Disrupted Cu homeostasis may affect antioxidant enzyme activity, alter oxidative stress in thyroid cells, and affect thyroid hormone synthesis and secretion (31, 32).

ALB, ALT, and ALP were retained simultaneously in the final model. Previous research has reported associations of GHT with liver function markers and neonatal birth weight, suggesting potential interactions among thyroid dysfunction, hepatic metabolic status, and pregnancy outcomes (33). Mechanistically, the liver participates in the synthesis of binding proteins, including albumin, thyroxine-binding globulin, and transthyretin, and contributes to peripheral conversion of T4 to T3. Thyroid hormones also regulate hepatic glucose and lipid metabolism, bile acid metabolism, and mitochondrial function (34). Therefore, the associations of ALB, ALT, and ALP with GHT risk in this study may reflect combined changes in protein nutritional reserve, hepatic metabolic adaptation, pregnancy-related physiological changes, and the thyroid axis. Because ALP may vary physiologically during pregnancy, its contribution should be interpreted with consideration of gestational timing. In the GW-adjusted sensitivity analysis, ALP remained the leading feature, whereas GW showed the lowest contribution, suggesting that the predictive role of ALP was not fully attributable to gestational week. These findings should not be directly interpreted as specific signals of liver disease.

Methodologically, this study compared 12 models under the same feature input, data partition, and preprocessing framework. Recent advances in machine learning and deep learning have supported computer-aided diagnosis and risk prediction across different medical fields, including image-based disease detection and endocrine disorder assessment. Yadav et al. developed an automatic computer-aided diagnosis tool for glaucoma detection using U-Net and CNN on retinal fundus images (35). Dhanka et al. reviewed advances in machine learning and deep learning for hormonal disorder diagnosis, including PCOS, thyroid disorders, and optimization techniques (36). Recent studies have also increasingly applied machine learning to pregnancy-related risk stratification. For example, Xing et al. developed GDMPredictor, a machine-learning tool for gestational diabetes mellitus risk assessment and treatment recommendation based on clinical and biochemical records (37). Compared with such broader gestational disease prediction tools, the present study focused on incident GHT and adopted a relatively parsimonious laboratory-marker model combined with SHAP-based interpretation to support risk stratification and individualized monitoring. Model performance was evaluated using discrimination, classification metrics, calibration, clinical net benefit, and residual distribution. The TRIPOD+AI statement emphasizes that clinical prediction models developed using regression or machine-learning methods should transparently report data sources, predictors, missing-data handling, model development, validation, and performance evaluation (38). PROBAST+AI further emphasizes assessment of risk of bias, applicability, and conditions for external generalization in prediction model studies (39). In obstetrics, LightGBM combined with SHAP has been used to predict sleep disorders during pregnancy and provided feature-level explanations (40). Studies predicting adverse outcomes in gestational diabetes mellitus also indicate that machine learning models may perform well in development datasets, but performance may decrease during external validation (41). Khan et al used interpretable machine learning to predict preterm birth in 3,509 pregnant women and emphasized that SHAP can support interpretation of individual predictions but cannot replace external validation (42). Therefore, the selection of LightGBM and the use of SHAP interpretation in this study are methodologically appropriate, whereas clinical implementation still requires further validation.

This study provides an interpretable auxiliary tool for GHT risk stratification at the first prenatal assessment. The model is not intended to replace TSH and FT4 testing or clinical diagnosis. It uses clinical laboratory markers to identify pregnant women at increased risk of subsequent GHT, thereby providing a reference for thyroid function retesting, nutritional assessment, and individualized follow-up. Similarly, Wang et al. showed, using an interpretable machine learning model for perinatal depression, that external validation and an interpretability framework may improve the usability of models in clinical risk screening (43). Studies on explainable artificial intelligence in medicine have also noted that methods such as SHAP can improve model transparency. However, SHAP results remain model-level associative contributions and cannot be directly interpreted as causal mechanisms (44). Therefore, the SHAP contributions of Zn, ALP, ALB, Cu, and other variables in this study should be interpreted as risk signals identified by the model rather than direct causal evidence for GHT.

This study has several limitations. First, this was a single-center retrospective study for prediction model development and internal validation; therefore, the generalizability of the model to other hospitals and populations remains uncertain. The observed event fraction reflected the outcome distribution of the analytic dataset and should not be interpreted as the population incidence of incident GHT. Second, outcome status was determined from available post-baseline thyroid function records and prenatal follow-up information. Because thyroid function retesting schedules and follow-up intensity were not fully standardized, outcome ascertainment bias may exist. Third, locally established pregnancy-specific or trimester-specific thyroid function reference intervals were unavailable, which may have introduced potential outcome misclassification. Fourth, although multiple imputation was used to handle missing data, residual bias related to the missing-data mechanism cannot be fully excluded. Fifth, some predictors, particularly trace elements and vitamin-related markers, may not be routinely measured in all prenatal care settings, which may limit direct model implementation in settings with different testing workflows. Finally, SHAP-based interpretation reflects model-derived associations rather than causal relationships. External prospective validation and, if necessary, local recalibration are required before clinical implementation.

## Conclusion

A LightGBM model using clinical laboratory markers showed balanced performance for predicting incident GHT. SHAP analysis identified Zn, ALP, ALB, Cu, vitE, ALT, Fe, Ca, and vitD as the leading contributors to model output. These results suggest that a multidimensional laboratory profile can characterize the risk of incident GHT. External prospective validation is needed before clinical implementation.

## Data Availability

The datasets presented in this article are not readily available because the datasets used and/or analyzed during the current study are not public-ly available due to institutional and patient privacy restrictions, but are available from the corresponding author on reasonable request and subject to institutional approval. Requests to access the datasets should be directed to Mengting Li: 1076302121@qq.com.
