# Supplementary material for: Development and validation of an interpretable machine learning model for predicting incident gestational hypothyroidism using clinical laboratory markers
Source: Front Med (Lausanne). 2026 Jul 1;13:1881157. doi: 10.3389/fmed.2026.1881157 (PMC13368916; doi:10.3389/fmed.2026.1881157)
Supplement: Supplementary file 1 [file Data_Sheet_1.docx]

**Supplementary Table S1. Missing data patterns before multiple imputation**

| **Variable** | **Missing n** | **Missing %** |
| --- | --- | --- |
| **P** | **17** | **4.18** |
| **LDH** | **14** | **3.44** |
| **Zn** | **14** | **3.44** |
| **vitD3** | **11** | **2.70** |
| **vitA** | **10** | **2.46** |
| **GLU** | **9** | **2.21** |
| **Cu** | **9** | **2.21** |
| **Fe** | **9** | **2.21** |
| **Ca** | **7** | **1.72** |
| **vitE** | **7** | **1.72** |
| **vitD** | **4** | **0.98** |

**Note: Missingness was assessed before multiple imputation. A total of 72 participants had at least one missing value, corresponding to 111 missing values in the original dataset. Variables not listed in the table had no missing values.**

**P, phosphorus; LDH, lactate dehydrogenase; Zn, zinc; vitD3, vitamin D3; vitA, vitamin A; GLU, glucose; Cu, copper; Fe, iron; Ca, calcium; vitE, vitamin E; vitD, vitamin D.**

**Supplementary Table S2. Quantitative calibration metrics of machine learning models in the training and internal validation sets**

| **Model** | **Training set** | | | **Internal validation set** | | |
| --- | --- | --- | --- | --- | --- | --- |
|  | **Brier score** | **Calibration intercept** | **Calibration slope** | **Brier score** | **Calibration intercept** | **Calibration slope** |
| **RandomForest** | **0.021** | **-0.014** | **26.783** | **0.138** | **0.064** | **2.227** |
| **SVM_Kernel** | **0.118** | **-0.066** | **1.090** | **0.120** | **-0.018** | **1.171** |
| **LogisticModel** | **0.132** | **0.000** | **1.000** | **0.119** | **0.149** | **1.237** |
| **NeighborMethod** | **0.157** | **-0.088** | **1.149** | **0.165** | **0.023** | **0.285** |
| **PLSModel** | **0.186** | **-0.230** | **3.058** | **0.177** | **-0.207** | **3.297** |
| **BoostingMethod** | **0.051** | **-0.031** | **2.658** | **0.125** | **0.214** | **1.096** |
| **NeuralNet** | **0.126** | **0.009** | **1.116** | **0.143** | **0.092** | **0.903** |
| **BayesMethod** | **0.132** | **-0.018** | **0.971** | **0.122** | **0.110** | **1.213** |
| **DiscriminantModel** | **0.132** | **-0.003** | **0.973** | **0.119** | **0.126** | **1.210** |
| **Lasso** | **0.134** | **0.000** | **1.380** | **0.124** | **0.102** | **1.713** |
| **AdaptiveBoosting** | **0.123** | **-0.224** | **1137.427** | **0.173** | **-0.196** | **4.039** |
| **LightGBM** | **0.077** | **0.022** | **2.198** | **0.131** | **0.096** | **1.040** |

SVM, support vector machine; PLS, partial least squares; Lasso, least absolute shrinkage and selection operator; LightGBM, light gradient boosting machine.

**Supplementary Table S3 Feature importance and discrimination of the GW-adjusted LightGBM model**

| **Feature** | **Gain** | **Cover** | **Frequency** |
| --- | --- | --- | --- |
| **ALP** | **0.195** | **0.171** | **0.139** |
| **Zn** | **0.162** | **0.154** | **0.132** |
| **vitE** | **0.109** | **0.081** | **0.096** |
| **ALB** | **0.093** | **0.109** | **0.106** |
| **ALT** | **0.093** | **0.092** | **0.111** |
| **Cu** | **0.085** | **0.074** | **0.078** |
| **Fe** | **0.084** | **0.097** | **0.094** |
| **vitD** | **0.081** | **0.089** | **0.118** |
| **Ca** | **0.078** | **0.107** | **0.092** |
| **GW** | **0.021** | **0.025** | **0.035** |

**The GW-adjusted LightGBM model was developed by adding gestational week to the final predictor set and using the same data partition and preprocessing workflow as the primary model. The model achieved an AUC of 0.977 in the training set and 0.915 in the internal validation set.**

**ALP, alkaline phosphatase; Zn, zinc; vitE, vitamin E; ALB, albumin; ALT, alanine aminotransferase; Cu, copper; Fe, iron; vitD, vitamin D; Ca, calcium; GW, gestational week; AUC, area under the receiver operating characteristic curve.**
